# Using individualized structural covariance networks to analyze the heterogeneity of cerebral small vessel disease with depressive states

**DOI:** 10.3389/fneur.2025.1541709

**Published:** 2025-04-08

**Authors:** Shiyu Zhang, Yue Chen, Hua Zhou, Zhong Zhao

**Affiliations:** ^1^The First People’s Hospital of Kunshan, Suzhou, China; ^2^Department of Neurology, The Affiliated Suzhou Hospital of Nanjing Medical University, Suzhou, Liaoning, China

**Keywords:** individualized differential structural covariance network, cerebral small vessel disease, depression, structural covariance network (SCN), heterogeneity

## Abstract

**Objectives:**

Cerebral small vessel disease (CSVD) is a heterogeneous cerebrovascular syndrome with a variety of pathological mechanisms and clinical manifestations. A majority of research have shown that CSVD is associated with reduced expression of structural covariance networks (SCNs), but most of these SCN studies based on the group-level, which limits their ability to reflect individual variations in heterogeneous diseases. The purpose of this study is to analyze the structural covariance aberrations in patients with cerebral small vessels by utilizing individualized differential structural covariance network (IDSCN) analysis to explore the differences in SCNs and depressive states at the individual-level.

**Methods:**

A total of 22 CSVD patients with depression (CSVD+D) and 34 healthy controls (HCs) were included in this study. IDSCNs were constructed for each subject based on regional gray matter volumes derived from their T1-weighted MRI images. The unpaired-sample t-test was used to compare the IDSCNs between the two groups to obtain the differential structural covariance edge and its distribution. Finally, correlation analyses were performed between the differential edge, the total CSVD imaging burden and Hamilton Rating Scale for Depression (HAMD) score.

**Results:**

(1) Compared with HCs, the CSVD+D patients exhibited heterogeneous distributions of differential structural covariance edge, with the differential edge located between the caudate nucleus and the cerebellum. (2) There was a significant positive correlation between the total CSVD imaging burden and HAMD scores in CSVD patients with depression (*r* = 0.692, *p* < 0.001).

**Conclusion:**

This study analyzed the IDSCNs between CSVD+D patients and HCs, which may indicate that the individual structural covariance aberrations between the caudate nucleus and cerebellum may contribute to depression in CSVD patients. Additionally, the higher total CSVD imaging burden of CSVD patients may indicate more severe depression. This finding suggests that early magnetic resonance imaging (MRI) assessment in CSVD patients may facilitate the early identification of depressive states and their severity in the near future.

## 1 Introduction

Cerebral small vessel disease (CSVD) is a highly heterogeneous cerebrovascular syndrome, which is a cluster of vascular diseases with distinct pathological backgrounds (1), including small arteries and their distal branches, microarterioles, capillaries, and small veins. The clinical manifestations of CSVD are complex and diverse, encompassing ischemic or hemorrhagic strokes, cognitive dysfunction, affective disorders, and gait abnormalities. Affective disorders are primarily characterized by depression, apathy, emotional instability, anxiety, and irritability, with depression being the most prominent feature (2). However, the clinical diagnosis and treatment of CSVD often lack specificity, and most of the current diagnosis relies on imaging examinations. With the advancement of multimodal neuroimaging, several well-recognized imaging markers of CSVD on MRI have been identified, including white matter hyperintensities (WMHs), cerebral microbleeds (CMBs), lacunes, enlarged perivascular spaces (EPVS), and brain atrophy. The correlation between these neuroimaging features and depressive symptoms has also been demonstrated. For example, some studies have shown that white matter microstructural damage and cerebral microbleeds are associated with depressive symptoms in elderly patients (3, 4).

Structural Covariance Network (SCN) is a network that reflects the synergistic changes between the morphological characteristics of different brain regions. It is based on the morphological characteristics of different brain regions extracted from magnetic resonance imaging (MRI) and reflects the coordinated development and synchronized maturation between brain regions (5). Compared with the effective connectivity network or functional connectivity network, SCNs reflect long-term changes and stability in brain structure, providing more stable and reliable measurements of brain network properties over extended periods. This not only enhances our understanding of the dynamic processes of brain structural changes but also offers unique insights into disease progression and the chronic effects on brain structure. However, most traditional studies of structural covariance networks are population-based, limiting their ability to identify individual differences in heterogeneous diseases like CSVD.

In 2021, Liu et al. (6) proposed a new method of network template perturbation to construct the individualized structural covariance network based only on T1-weighted images. This approach allows for the exploration of structural morphological aberrations between brain regions at the individual level and can potentially identify subtypes with unique neuroanatomical patterns in heterogeneous disorders, thereby better elucidating individual-level heterogeneity.

In our study, we first obtained the IDSCN of each subject using individualized difference structured covariance network (IDSCN) analysis and identified differential edges by comparing healthy controls with the CSVD+D group. Subsequently, we correlated the differential edges and the total CSVD imaging burden with HAMD scores.

This study aims to assess the presence and severity of depressive states in CSVD patients through early individual-level MRI assessment, thereby providing patients with timely psychological counseling and pharmacological interventions.

## 2 Materials and methods

### 2.1 General information

#### 2.1.1 Research target

A total of 56 subjects attending the Department of Neurology at the Affiliated Suzhou Hospital, Nanjing Medical University were enrolled in this study, including 22 CSVD patients with depressive symptoms (CSVD+D) and 34 healthy controls (HCs). All subjects were Han Chinese and right-handed. The study was approved by the Ethics Committee of the Affiliated Suzhou Hospital, Nanjing Medical University (K2017034). Written informed consent was obtained from all subjects prior to enrollment.

#### 2.1.2 Inclusion and exclusion criteria

##### 2.1.2.1 Inclusion criteria

The inclusion criteria for HC were as follows: (1) individuals aging 50–80 years; (2) education ≥6 years; (3) the ability to cooperate with neuropsychological scale assessment and meet the following scores: HAMD ≤7, HAMA <7, MMSE ≥27, and MoCA ≥25.

The inclusion criteria for CSVD+D were as follows: (1) individuals aging 50–80 years; (2) education ≥6 years; (3) presence of typical CSVD imaging features (e.g., white matter hyperintensities, lacunae, microhemorrhages, or enlarged perivascular spaces, etc.); (4) the ability to cooperate with neuropsychological scale assessment and meet the following score: HAMD >7.

##### 2.1.2.2 Exclusion criteria

Exclusion criteria for all subjects were as follows: (1) presence of subcortical nonlacunar infarcts or cortical infarcts >1.5 cm on MRI; (2) intracranial and extracranial vascular stenosis ≥50%; (3) cardiogenic embolism; (4) traumatic, space-occupying, or inflammatory disorders of the central nervous system (CNS); (5) hepatic encephalopathy, renal encephalopathy, or hypoxic–ischemic encephalopathies; (6) severe anxiety, depressive disorders, or other severe mental disorders; (7) cognitive impairment due to other diseases (e.g., Alzheimer’s disease); (8) MRI contraindications.

#### 2.1.3 Diagnostic criteria and scale assessment

The diagnostic criteria for Cerebral Small Vessel Disease (CSVD) are primarily based on radiological features and clinical manifestations (7). Radiological features include white matter hyperintensities, lacunes, cerebral microbleeds, and enlarged perivascular spaces. Typical clinical manifestations include cognitive decline, gait disturbances, emotional disorders, and urinary or fecal incontinence.

The diagnosis of depressive disorders in this study was mainly based on the WHO International Classification of Diseases, Eleventh Edition (ICD-11) (8) definition of a depressive episode, with the main symptoms including depressed mood, reduced interest and pleasure in activities. Associated symptoms may also be present, such as difficulty concentrating, feelings of worthlessness, excessive guilt, hopelessness, recurrent thoughts of death or suicide, changes in sleep or appetite, psychomotor retardation or agitation, and decreased energy or fatigue. These symptoms were present most of the day and persisted for at least 2 weeks. In major depressive episodes, the majority or nearly all symptoms, including core and associated symptoms, are prominently manifested. Patients experience significant functional impairment and are typically unable to function normally in important areas such as personal, family, social, and occupational domains. A HAMD-17 score of ≥24 is classified as severe depression (9).

All subjects were assessed with a series of neuropsychological scales including the 17-item Hamilton Rating Scale for Depression (HAMD-17) (9), the Mini-Mental State Examination (MMSE) (10), the Montreal Cognitive Assessment (MoCA) (11). The severity of depression in CSVD patients was assessed based on HAMD scale scores, with all patients in the CSVD+D group having HAMD scores >7 (9). All patients enrolled in the CSVD+D group did not have significant cognitive dysfunction.

### 2.2 Data acquisition and imaging marker evaluation

In this study, all patients underwent 3.0 T MRI scanning (Shanghai United Imaging), with the whole-brain sequences including T1-weighted 3D imaging, T2-weighted imaging, fluid-attenuated inversion recovery (T2-FLAIR) imaging, and susceptibility-weighted imaging (SWI). The acquisition parameters for T1-weighted 3D imaging were as follows: echo repetition time (TR) = 2,773 ms, echo time (TE) = 69 ms, slice thickness = 2.0 mm, slice spacing = 10.0 mm, flip angle = 120°, scanning field size = 120 mm x 120 mm, The acquisition parameters of susceptibility-weighted imaging were as follows: TR = 30.2 ms, TE = 20 ms, flip angle = 15°, slice thickness = 2.0 mm, slice spacing = 2.0 mm, scanning field size = 224 × 190 mm.

The lesion prediction algorithm (LPA) (11) from Lesion Segmentation Toolbox version 3.0.0 (LST)^1^ in SPM was used for segmentation calculation of white matter hyperintensities on T2-FLAIR images. Additionally, two trained evaluators counted CMBs, EPVS, WMHs, and lacunae according to the diagnostic criteria of the Neuroimaging Vascular Alteration Criteria (12) based on T1-weighted imaging, T2-weighted imaging, fluid-attenuated inversion recovery imaging, and susceptibility-weighted imaging. CMBs are defined as an area of signal cavity with a diameter of 2–5 mm (rarely up to 10 mm) and a circular or elliptical shape on SWI. EPVS are defined as fluid-filled cavities surrounding arteries, arterioles, veins, and venules as they course from the subarachnoid space through the brain parenchyma. WMHs are characterized as high signal on T2-weighted imaging and fluid-attenuated inversion recovery imaging sequences. Lacunae are defined as round or ovoid fluid-filled cavities with cerebrospinal fluid-like signals between 3 and 15 mm in diameter, which located in the white matter, basal ganglia, or thalamus (12). The total MRI burden of CSVD (13) was derived by calculating neuroimaging markers including lacunae, cerebral microbleeds (CMBs), white matter hyperintensities (WMHs), and enlarged perivascular spaces (EPVS), with one point for each marker present, yielding a maximum score of 4.

### 2.3 Experimental procedures

#### 2.3.1 Voxel-based morphometry analysis

Voxel-based morphometry (14) was performed on the 3D-T1-weighted images of all subjects using the CAT12 toolbox in SPM12.^2^ The main components include: (1) Spatial normalization: The MRI images of all subjects were aligned to a spatial standard template to correct for differences in overall brain morphology and anatomical location. (2) Bias field correction: Reduced the brightness differences of the same tissue to facilitate subsequent tissue segmentation. (3) Segmentation of brain tissue: The 3D-T1 weighted images were segmented into gray matter, white matter, and non-cerebral voxels (cerebrospinal fluid). (4) Modulation: The deformation field formed by spatial normalization was applied to the segmentation result to preserve the volume of the original individual tissue volume (voxel size = 1.5 × 1.5 × 1.5 mm). (5) Smoothing: Noise generated by the above process was removed to avoid false-positive statistical results. (A Gaussian kernel with a maximum radius of 6 mm was used for smoothing). Finally, the total intracranial volume (TIV) of each subject was also calculated to control for covariates in the statistical analysis.

The AAL116 template was used to divide the whole brain into 116 brain regions (90 cerebral and 26 cerebellar regions) for the subsequent construction of individualized structural covariance networks.

#### 2.3.2 Constructing individualized differential structure covariance networks

**Figure 1:**
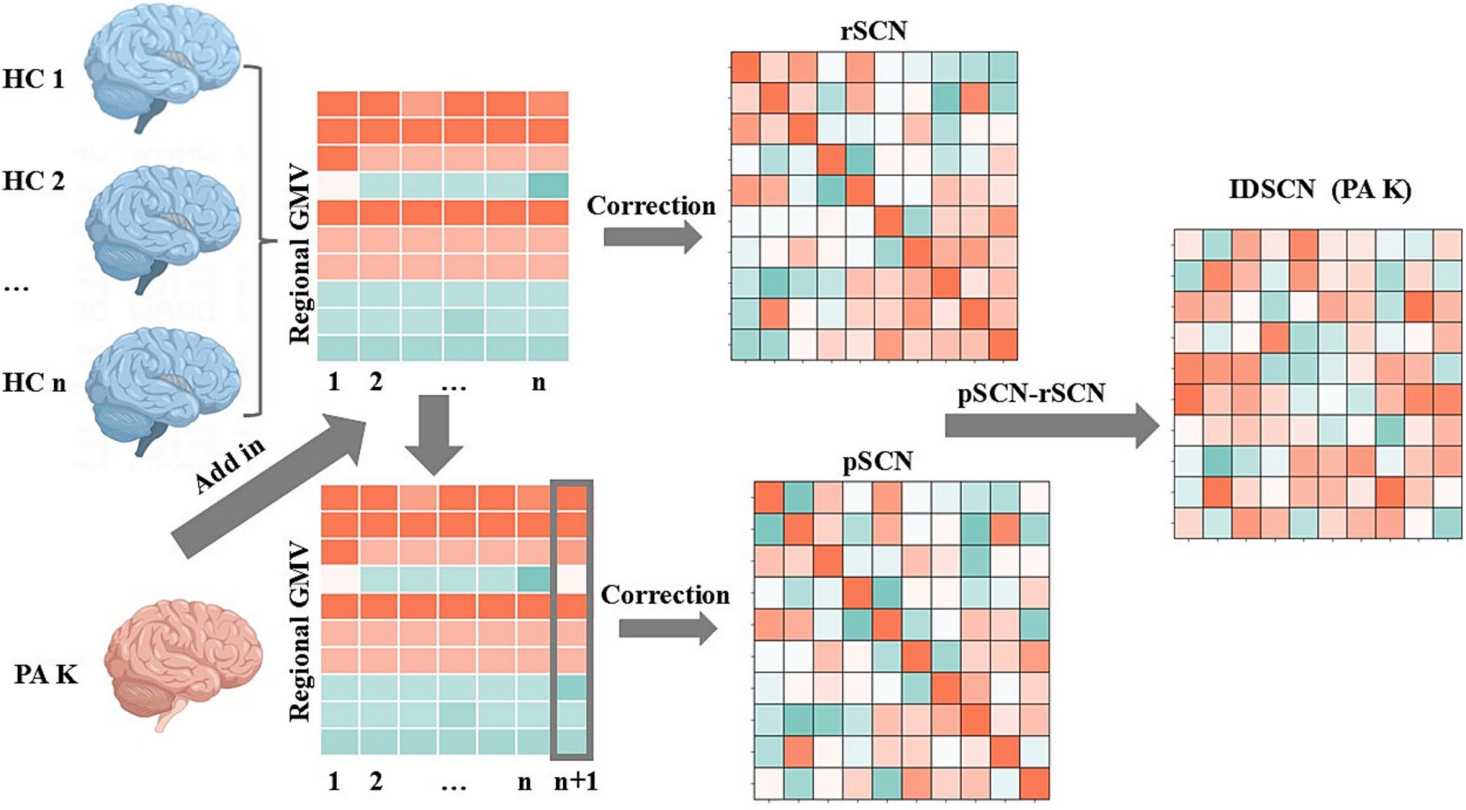
Flowchart of individualized differential structural covariance network construction. This figure illustrates the workflow for constructing an IDSCN of PA K. Initially, a reference structural covariance network (rSCN) at the group level was established using healthy controls (HCs). On this basis, a perturbed structural covariance network (pSCN) was constructed by adding patient K (n HCs and 1 PA K). The IDSCN of patient K was calculated as the difference between rSCN and pSCN.

(1) Construction of a reference structural covariance network (rSCN): n healthy controls in the HC group were used to construct a reference structural covariance network, which was achieved by calculating the correlation between gray matter volumes of brain regions, and each edge in the network was obtained by calculating the Pearson’s correlation coefficients (PCC) between gray matter volumes of pairs of brain regions. Age, sex, education, and total intracranial volume were included as covariates (Figure 1).
(2) Construction of a perturbed structural covariance network (pSCN): A random patient K was added to the HC group to form a new population of n + 1 individuals, and a new structural covariance network was constructed in the same manner. Since the addition of patient K perturbed the original population, the newly constructed network was termed a perturbed structural covariance network.
(3) Construction of the individualized structural covariance network (IDSCN): The difference between the perturbed structural covariance network and the reference structural covariance network was defined as ΔSCN (ΔSCN = pSCN − rSCN), which represents the IDSCN of patient K. ΔSCN = pSCN − rSCN can be interpreted as the difference in Pearson correlation coefficients (PCC) between corresponding edges of pSCN and rSCN. Further details are provided in the study by Liu et al. (15). The IDSCN was constructed for each patient sequentially. The same methodology, replacing patient K with a randomized subject from a healthy control, was used to obtain an individualized structural covariance network for each healthy subject.
(4) Calculation of edge weights in the IDSCN: The edges of the IDSCN represent the change in the covariance of the paired brain regions in the gray matter volume between the perturbed group and the reference group after adding patient K. Based on the AAL116 template, we obtained 6,670 edges across 116 brain regions. According to Liu et al. (15), the difference between pSCN and rSCN (ΔSCN) follows a symmetric “volcano distribution” with tails similar to those of a normal distribution. The Z-score of ΔSCN can be calculated using the Z-test, and the weight of each edge can be expressed as the Z-score calculated by the following formula:

$$Z=\frac{\mathrm{\Delta SCN}}{\left(1-{\mathrm{rSCN}}^{2}\right)/\left(n-1\right)}$$

Positive *Z*-values indicate that the structural covariance edges are stronger in patient K than in the healthy population. Negative *Z*-values indicate that the structural covariance edges are weaker in patient K than in the healthy population. The *p*-values of the network edges were obtained from the Z-scores. In this study, we finally obtain the differential structural covariance edge for each patient that were significantly different from the reference network. (Bonferroni correction, *p* < 0.05).

### 2.4 Statistical analysis

In this study, two-sample unpaired T-test was used for difference analysis of measurement data (including age, MMSE score, MoCA score, HAMD score and TIV) in demographic data between the two groups. Chi-square test was used for difference analysis of categorical data (including gender, hypertension, diabetes and hyperlipidemia) in demographic data between the two groups. All the above statistical analyses were performed using SPSS, version 26.0.^3^

Differences in network edges between the two groups were compared using a two-sample unpaired T-test in a graph theory-based toolbox for brain network analysis, Gretna (NITRC: GRETNA: Tool/Resource Info). Bonferroni correction was applied (*p* < 0.05 considered statistically significant).

Finally, Spearman’s correlation was used to perform correlation analysis of differential edges, total MRI burden of Cerebral Small Vessel Disease, and HAMD scale scores, respectively. Two Spearman correlation analyses were performed. Bonferroni correction was applied (*p* < 0.005 considered statistically significant).

## 3 Results

### 3.1 Demographic information

Table 1, display the demographic characteristics, vascular risk factors, scale scores, and imaging markers of two groups. As indicated in Table 1, there were no significant differences in demographic characteristics, vascular risk factors, and cognitive scale scores (MMSE and MoCA) between the two groups (*p* > 0.05). HAMD scale scores were significantly higher in CSVD patients with depressive disorders than in healthy controls (*p* < 0.05).

**Table 1:** Demographic characteristics, scale scores, and imaging markers of the study participants.

	HC (*n* = 34)	CSVD+D (*n* = 22)	*P*
Gender, (M/F)	16/18	12/10	0.584^b^
Age, (mean ± SD)	64.9 ± 6.4	67.9 ± 8.8	0.182^a^
MMSE score, (mean ± SD)	29.0 ± 1.1	28.6 ± 1.3	0.119^a^
MoCA score, (mean ± SD)	27.7 ± 1.9	26.8 ± 1.6	0.084^a^
HAMD score, (mean ± SD)	0.3 ± 0.6	11.3 ± 4.7	<0.001^a^
WMH, (mean ± SD)	–	12.2 ± 13.2	–
LI, (mean ± SD)	–	3.8 ± 3.2	–
CMB, (mean ± SD)	–	1.6 ± 3.7	–
EPVS, n(%)	–	11 (50)	–
TIV, (mean ± SD)	1434.6 ± 132.6	1466.4 ± 139.9	0.394^a^
Hypertension, n(%)	17 (50)	12 (54)	0.740^b^
Diabetes, n(%)	6 (18)	7 (32)	0.220^b^
Hyperlipidemia, n(%)	10 (29)	7 (32)	0.848^b^

a *p-values computed implementing the two-sample unpaired t-test.*

b *p-values computed implementing the χ2-test.*

HC, healthy control group; CSVD+D, CSVD (cerebral small vessel disease) patients with depressive states; MMSE: Mini-mental Status Examination Scale; MoCA, Montreal Cognitive Assessment Scale; HAMD, Hamilton Depression Scale; WMH. white matter hyperintensities; LI, lacunae; CMB, cerebral microbleeds; EPVS, enlarged perivascular spaces; TIV, total intracranial volume. Outcomes indicate the mean ± SD or number of candidates (%).

### 3.2 Distribution of differential structural covariance edges between groups

**Figure 2:**
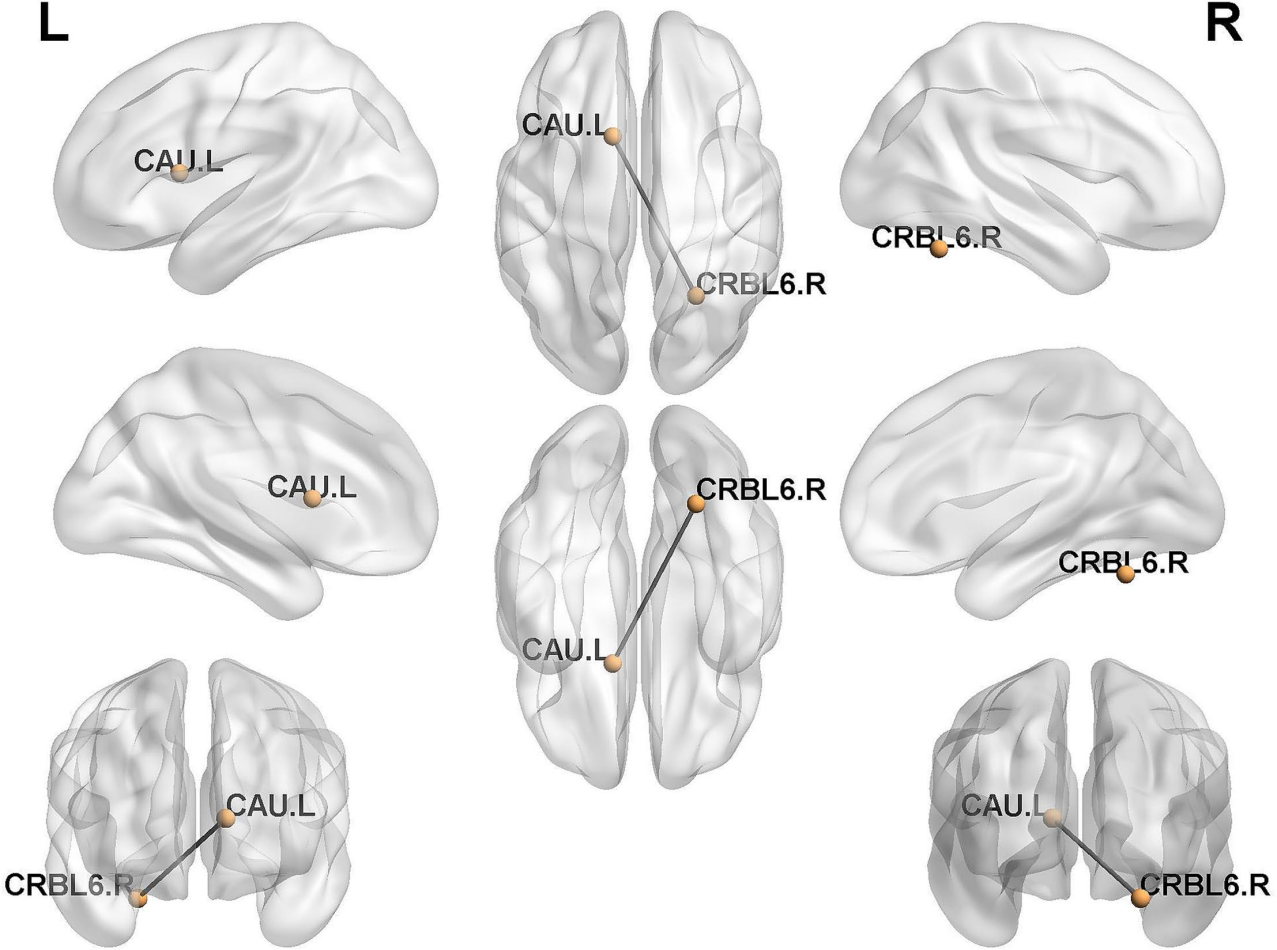
Distribution of differential structural covariance edges between groups. CRBL6.R, Cerebelum_6_R; CAU.L, Caudate_L.

In this study, the individualized structural covariance networks of all subjects in HC and CSVD+D groups were obtained. Subsequently, differences in network edges between the two groups were assessed using a two-sample unpaired *t*-test with Bonferroni correction. One significantly differential structural covariance edge was finally identified (*p* < 0.05). This differential edge was distributed between the caudate nucleus and the cerebellum (Figure 2).

### 3.3 Correlation analysis of differential edge and total MRI burden of CSVD with degree of depression

**Figure 3:**
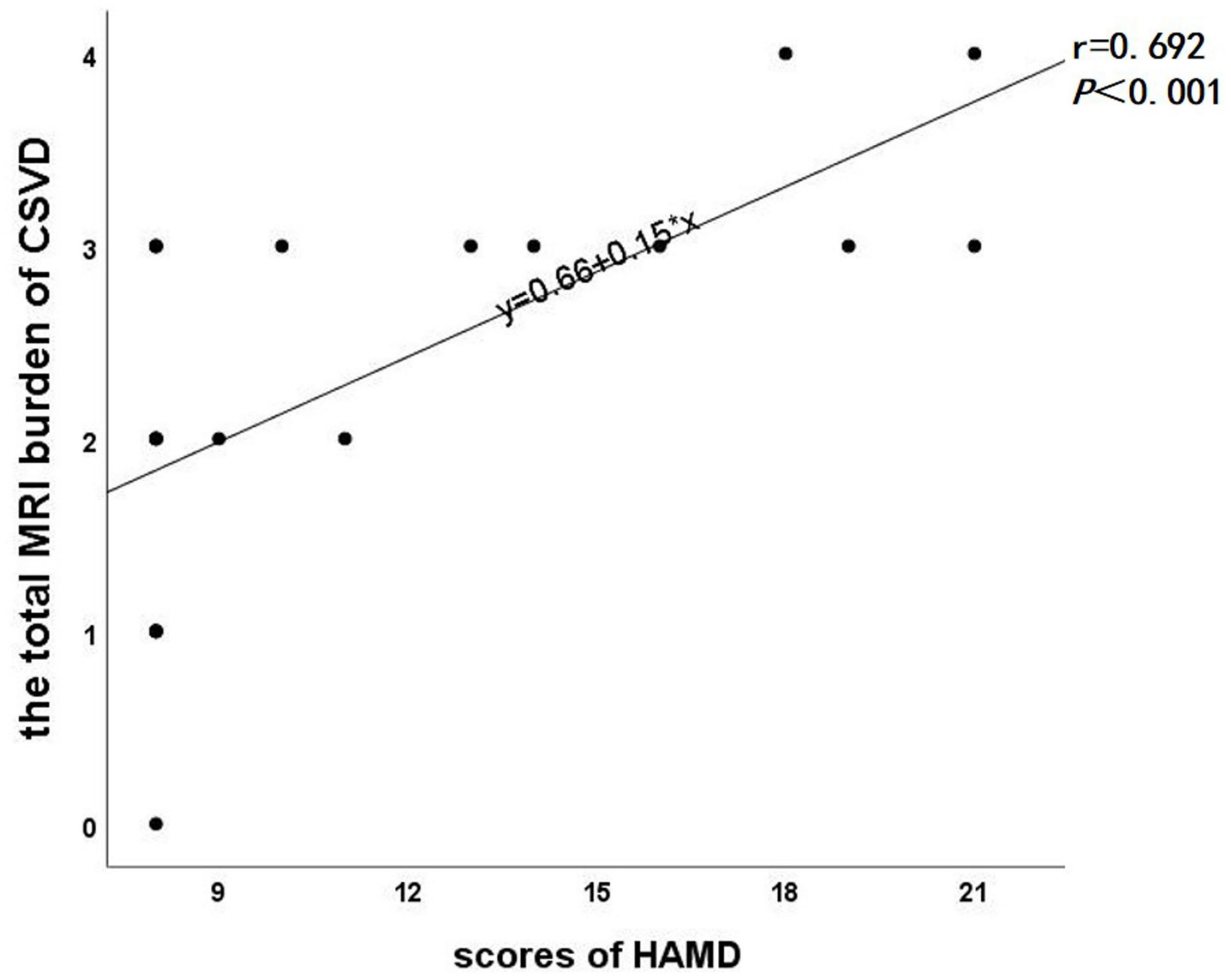
Scatter plot of correlation between total MRI burden of CSVD and HAMD scale scores.

In the subsequent correlation analysis, a significant positive correlation was observed between the total MRI burden of CSVD and HAMD scale scores (*r* = 0.692, *p* < 0.001, Figure 3). However, no significant correlation was found between the differential edge and HAMD scale scores (*P* > 0.05).

## 4 Discussion

In this study, we obtained individualized structural covariance networks for each subjects in both group of HC and CSVD+D. Compared with healthy controls, CSVD patients with depressive states exhibited the aberrations of covariance in network edge, with the differential edge distributing between the striatum and the cerebellum.

Previous studies have indicated that there were aberrations of structural covariance in CSVD patients’ brain network (16, 17). However, these studies mostly constructed structural covariance networks at the group level, and such aberrations were not representative of individual differences. Subsequently, Han et al. (18) found that patients with depression had reduced correlated edges in the subcortical cerebellar network while exploring their individualized structural covariance networks. Similarly, our study found that CSVD patients with depressive states had a differential edge distributed between the caudate nucleus and the cerebellum, based on individual-level analysis. The caudate nucleus, located in the ventral striatum, is the largest component of the basal ganglia and, together with the cerebellum, forms an important part of the subcortical cerebellar network.

In terms of network connectivity, the basal ganglia and cerebellum play crucial roles in brain network connectivity and have significant emotional impacts. Previous studies have found that the basal ganglia, cerebellum, and cerebral cortex are interconnected and form an integrated network (19). The basal ganglia and cerebellum communicated not only with cerebral cortex through discrete trans-thalamic loops and indirectly via cortical projections, but also directly through subcortical pathways. Moreover, altered connectivity between nodes in such brain networks can affect emotion. For example, Baek et al. (20) found that cerebellar neurons projecting from the dentate nucleus to the basal ganglia mediate depressive-like behavior in mice. Jackson and Bernard (21) demonstrated that cerebellar and basal ganglia motor networks predict motivation and symptom of depression.

With respect to the molecular level, previous transcriptome researches on the striatum have indicated that dysfunction of dopaminergic synapses, GABAergic synapses and neurotransmitter synthesis are associated with the pathogenesis of depression (22, 23). Studies on rat model of post-stroke depression have also demonstrated that (24, 25) the expression of brain-derived neurotrophic factor (BDNF) and tyrosine kinase receptor B (TrkB) in cerebellum and amygdala is significantly reduced. However, previous studies have focused on single brain regions, thereby ignoring the systemic metabolic alterations that occur in multiple brain regions in depressive behaviors. Xu et al. (26) found that chronic stress may induce depressive behavior by disrupting the glutamine-glutamate-GABA (Gln-Glu-GABA) cycle in the striatum, hippocampus, and cerebellum based on the metabolomics study of multiple brain regions. A common significantly down-regulated differential metabolite, L-glutamine, was present between striatum and cerebellum. Furthermore, the disturbed shared pathways, enriched metabolite sets, and metabolic pathways between the striatum and cerebellum all indicate significant alterations in glutamate and glutamine metabolism in these two brain regions. It is hypothesized that the combined effects of dysregulation in the glutamatergic system between the striatum and the cerebellum may be critical in the pathogenesis of depression.

Further genetic analyses revealed the presence of differential genes associated with depressive-like behavior in the striatum, hippocampus, and cerebellum (26). *Differential genes in the* striatum *(GS) were significantly* correlated with cerebellar differential genes *(GS, GDH, and ABAT).* This suggests that the striatum and the cerebellum may be highly cooperative in the physiological mechanisms underlying the development of depressive-like behavior.

In a subsequent correlation analysis, we found a significant correlation between the total MRI burden of CSVD and HAMD scale scores. Patients with CSVD often present with multiple imaging markers simultaneously, making the assessment of total MRI burden of CSVD more clinically relevant (13). Zhou et al. (27) revealed that the total MRI burden of CSVD can predict post-stroke depression more accurately, which supports our findings. This may be due to the damage of white matter fiber tracts in CSVD patients with depressive states, which disrupts the connectivity of the brain’s structural network and leads to poorer emotional outcomes (28). No significant correlation was found between the resulting network differential structural covariance edge and HAMD scale scores. This may be attributed to the fact that cerebral small vessel disease and depressive states are complex and heterogeneous disorders with a wide variety of etiologies and manifestations, and it is possible that only the differential edges for a specific type or etiology of CSVD are significantly associated with depressive states. There is also the influence of confounding factors, such as small sample sizes, medication use (e.g., Chinese patent medicines for improving cerebrovascular diseases and antidepressants), and other comorbidities.

This study has some limitations, the sample size of the experimental group was small due to time constraints, and the results were not validated with another dataset. Second, this study was cross-sectional, and a causal relationship between depressive state and aberrations in the structural covariance between brain regions could not be established.

In summary, previous studies have already examined population-level structural covariance networks in CSVD patients and individual-level structural covariance networks in depressed patients. This study represents the first individualized structural covariance network analysis of CSVD patients with depressive states. The results suggest that individual-level structural covariance aberrations between the cerebellum and the caudate nucleus may predict a higher propensity for depression in CSVD patients.

## Data Availability

The raw data supporting the conclusions of this article will be made available by the authors, without undue reservation.

## Footnotes

^1^ www.statistical-modeling.de/LST.html

^2^ http://dbm.neuro.uni-jena.de/cat12/

^3^ http://www.spss.com/
